# Methane and Carbon Dioxide Hydrate Formation and Dissociation in Presence of a Pure Quartz Porous Framework Impregnated with CuSn12 Metallic Powder: An Experimental Report

**DOI:** 10.3390/ma14175115

**Published:** 2021-09-06

**Authors:** Alberto Maria Gambelli, Giulia Stornelli, Andrea Di Schino, Federico Rossi

**Affiliations:** 1 Engineering Department, University of Perugia, Via G. Duranti 93, 06125 Perugia, Italy; federico.rossi@unipg.it; 2 Industrial Engineering Department, University of Rome “Tor Vergata”, Via del Politecnico 1, 00133 Rome, Italy; giulia.stornelli@students.uniroma2.eu

**Keywords:** natural gas hydrate, carbon dioxide capture, hydrate equilibrium, chemical promoters, CuSn12 metallic powder

## Abstract

Hydrate formation and dissociation processes were carried out in the presence of a pure quartz porous medium impregnated with a metallic powder made with a CuSn12 alloy. Experiments were firstly made in the absence of that powder; then, different concentrations were added to the porous medium: 4.23 wt.%, 18.01 wt.%, and 30.66 wt.%. Then, the hydrate dissociation values were compared with those present in the literature. The porous medium was found to act as an inhibitor in the presence of carbon dioxide, while it did not alter methane hydrate, whose formation proceeded similarly to the ideal trend. The addition of CuSn12 promoted the process significantly. In particular, in concentrations of up to 18.01 wt.%, CO_2_ hydrate formed at milder conditions until it moved below the ideal equilibrium curve. For methane, the addition of 30.66 wt.% of powder significantly reduced the pressure required to form hydrate, but in every case, dissociation values remained below the ideal equilibrium curve.

## 1. Introduction

Natural gas hydrate (NGH) is an ice-like solid compound composed by a crystalline structure based on water molecules, which contains natural gas molecules [1]. Hydrate compounds were discovered during the 18th century when, in 1778, Sr. Joseph Priestly made cold experiments in his laboratory in Birmingham and discovered that if temperature was kept close to the ice point, but in any case higher than this value, a mixture containing water and vitriolic air (SO_2_) was able to pass from the liquid to the solid phase. Starting from its discovery, the history of gas hydrate addressed three different periods. During the first period, hydrate compounds were simply considered a scientific curiosity; thus, scientific production remained particularly contained. Since 1934, due to its capability of causing gas pipelines blockage, gas hydrate started being studied with the aim of preventing its formation and avoiding the consequent issues for the natural gas industry. Finally, in the mid-1960s, scientific interest on gas hydrate rose due to the possibility of making it a new potential energy source. Just in this period, the first natural reservoirs were discovered and the first estimations about the amount of natural gas contained in those deposits were made. To date, these estimates still show some differences between them, but in all cases, the quantity of natural gas present in hydrate was found to be enough to produce more than twice the energy that can still be produced from all conventional energy sources put together [2,3]. NGH reservoirs are mainly sited in offshore sediments (≈97%) and in permafrost (≈3%). The most significant sites were found in the South China Sea, Japan Sea, Indian Ocean, Gulf of Mexico, Bearing Strait, and Seas of Korea, while regarding permafrost reservoirs, numerous findings were made in Alaska, the Mackenzie Delta, Siberia, and the Qinghai–Tibet Plateau [4].

Moreover, natural gas hydrate may consist of a carbon neutral energy source due to the possibility of recovering methane contained in water cages by replacing it with an equal number of carbon dioxide molecules. Energy-consuming countries such as America, China, Canada, and Japan carried out several efforts to make NGH reservoirs’ exploitation feasible both in offshore and in onshore sites. Unfortunately, hydrate exploitation is nowadays far from becoming attractive for industrial large-scale applications because of unsolved economic and environmental issues. Regarding this latter argument, it was established that the strength and rheology characteristics of natural gas hydrate are over 20 times higher than those of ice and, with the lowering of temperature, hydrate becomes more and more cohesive [5]. In sediments that are contained, hydrate compounds act as a solid skeleton of frame sediments and cement sediment particles [6,7]. Natural gas recovery means water cages dissociation, with a consequent loss of shear strength and instability of the well [8,9]. In addition, gas deriving from hydrate dissociation produces an increase in sand and rocks’ pore pressure, thus causing stress, failures, and the deformation of sediments [10]. Mechanical deformation of hydrated gas sediment strongly affects the mechanical stability of the whole reservoirs and may also impact the assessment of the gas yield from hydrate deposits [11,12]. As mentioned above, economic issues actually represent the most limiting factor. Intervening at extremely elevated depths, difficulties in gas diffusion through adjacent hydrate layers of the deposit and methane hydrate re-formation are some of the main reasons that make the process immature for industrial large-scale applications. In the last few decades, different techniques have been performed to promote natural gas recovery from NGH reservoirs; among them, the most effective and diffused are depressurization [13,14], thermal stimulation [15,16], and chemical inhibitor injection [17]. The first two techniques are based on modifying the local thermodynamic conditions in order to make them unfeasible for hydrate stability, thus causing its dissociation. As the name suggests, depressurization consists of lowering the local pressure while keeping temperature constant [18]. Conversely, with thermal stimulation, the local temperature is increased, while pressure remains constant [19]. Currently, depressurization and thermal stimulation are the most effective strategies; especially if coupled together, they assume the most elevated energy produced/energy spent ratio [20].

Chemical inhibitor injection is carried out by injecting chemical additives capable of shifting hydrate equilibrium to more severe thermodynamic conditions in order to make its permanence unfeasible at the local pressure–temperature values [17]. Currently, two major groups of chemical additives are used: thermodynamic and low-dosage hydrate inhibitors [21]. Thermodynamic inhibitors (THIs) are able to form hydrogen bonds with the adjacent water molecules and consequently lower the activity of water in hydrate formation. In turn, low-dosage hydrate inhibitors (LDHIs) are divided in kinetic inhibitors (KHIs) and anti-agglomerants (AAs). During hydrate formation, LDHIs allow extending the induction time and also the growth rate, thus lowering its formation. The most widespread thermodynamic inhibitors include methanol, glycols, and salts, while significant examples of LDHIs are polyvinylpyrrolidone and poly-N-vinylcaprolactam [21].

The adoption of chemical inhibitors in NGH reservoirs is considered prohibitive due to their elevated cost and even their high impact on the environment. The second issue may be partially solved by using amino acids such as carboxylic acid (-COOH) and an amine group (-NH_2_). Amino acids are biologically organic compounds and were found to be less harmful to the environment, thanks to their biodegradability [22].

Recently, the possibility of coupling chemical inhibitors injection with other strategies, i.e., depressurization, was proposed and experimentally investigated [23]; however, the overall feasibility of using such compounds has not been confirmed yet.

The present work deals with gas hydrate formation, with methane and carbon dioxide as guest molecules, in pure quartz sand and with the addition of a metallic CuSn12 powder. Tests were realized in a lab-scale reactor, and different powder concentrations were used. Experiments were carried out in order to establish whether a specific metallic powder is able to intervene on hydrate formation and dissociation conditions and, in a positive case, define its effect.

Hydrate compounds were firstly formed and then dissociated in order to produce the respective equilibrium values, which have been consequently compared with values present in the literature about CH_4_ and CO_2_ hydrate equilibrium. To the best of our knowledge, in the literature, there is currently a substantial lack of equilibrium values in the presence of that compound, while similar information could be extremely useful to better understand the interaction mechanism of metallic powders with hydrate formation and for potential practical applications. In particular, the present paper aimed to initially investigate the potentialities of realizing tanks and cylinders, having an internal high-porous metallic lattice, via 3D print technologies, in order to improve gas storage efficiency, thus increasing the energy density per unit of volume.

## 2. Materials and Methods

### 2.1. Experimental Apparatus

The lab-scale experimental apparatus used in this work to perform hydrate formation and dissociation has been already used in previous research, and a detailed description of it is present elsewhere in the literature [21,24]. The reactor consists of a 316SS cylindrical chamber, with an internal volume equal to 949 cm^3^ (diameter 7.3 cm and height 22.1 cm). Such material is chosen following its peculiar corrosion resistance also in severe applications [25,26,27]. More detailed information about geometry is provided in Table 1.

The extreme surfaces are closed with two flanges, and an equal number of spiro-metallic gaskets (model DN8U PN 10/40 316-FG C8 OR) were inserted to avoid any gas leakage. Gas injection may occur both from the upper and the lower flange. Usually, when replacement tests are performed, methane is injected from the bottom, while carbon dioxide is injected from the top. Here, both gases have been alternatively inserted from the bottom in order to guarantee a better diffusion of gaseous molecules inside sand pores.

**Figure 1:**
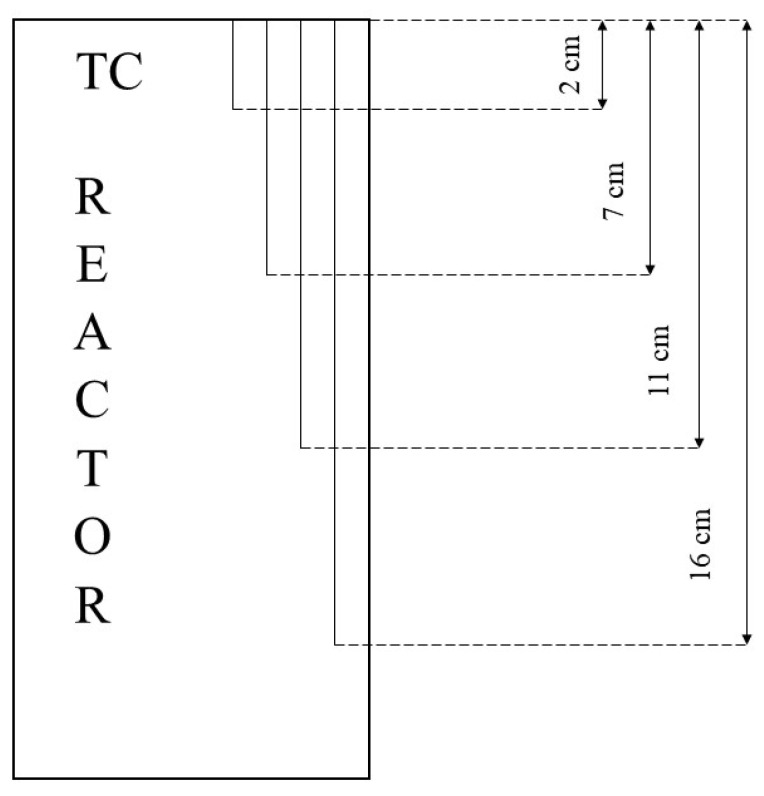
Thermocouples placement inside the reactor.

Temperature was monitored with four Type K thermocouples, having class accuracy 1 and positioned at four different depths, as shown in Figure 1.

**Figure 2:**
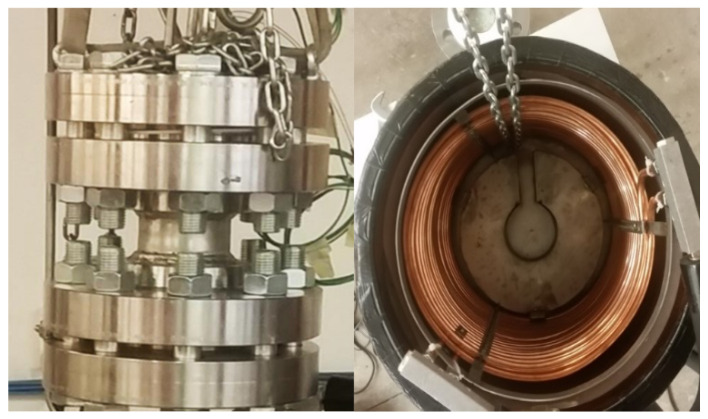

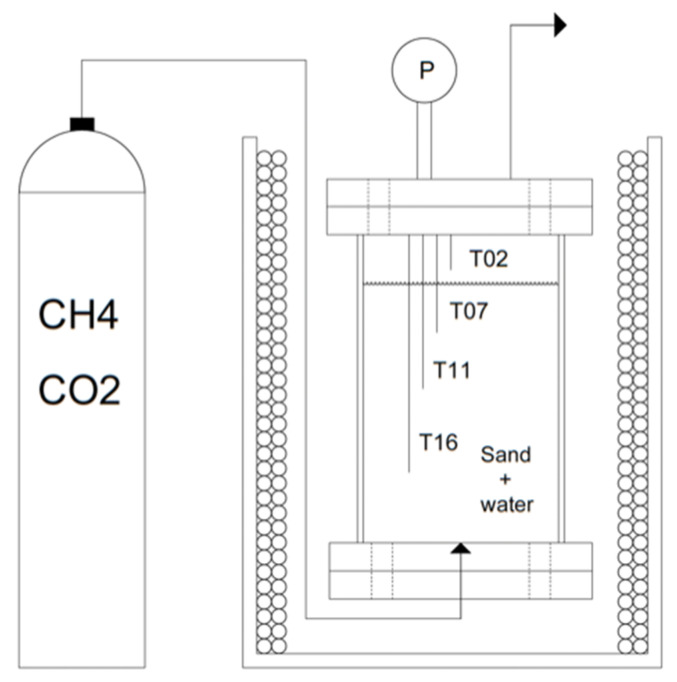
Picture of the lab-scale reactor (at **left**), the thermostatic bath in which it is inserted (at **right**), and scheme of the whole experimental apparatus (**below**).

Thermocouples have been named as the respective depths: T02, T07, T11, and T16. The temperature-monitoring system was established according to Wang et al. (2017), Yuan et al. (2012), and Yin et al. (2019) [28,29,30]. Temperature was controlled and varied from the external by inserting the reactor in a thermostatic bath directly connected with a chiller, model GC-LT. Figure 2 shows the 316SS reactor and the thermostatic bath internally equipped with a double copper coil to allow heat exchange between the bath and the refrigerating fluid (glycol), together with a scheme of the completely assembled experimental apparatus.

Pressure was measured by using a digital manometer, model MAN-SD, having an accuracy of ±0.5%. All sensors were connected to the Labview software with a data acquisition system manufactured by a National Instrument used for monitoring and recording data from sensors.

### 2.2. Materials

Ultra-High Purity (UHP) methane and carbon dioxide, with a purity respectively equal to 99.997% and 99.999%, were used for gas hydrate formation in addition to pure demineralized water and sand. This latter compound consists of pure quartz spheres, having diameter equal to 100 μm. The grain porosity is 34% and was measured with a porosimeter, model Thermo scientific Pascal 140. More in depth, the reactor was filled with 744 cm^3^ of sand and 236 cm^3^ of water; the remaining space was kept free for gas injection. In addition to those compounds, also a metallic CuSn12 powder was used, whose specifications are described in the next section.

#### CuSn12 Powder

**Figure 3:**
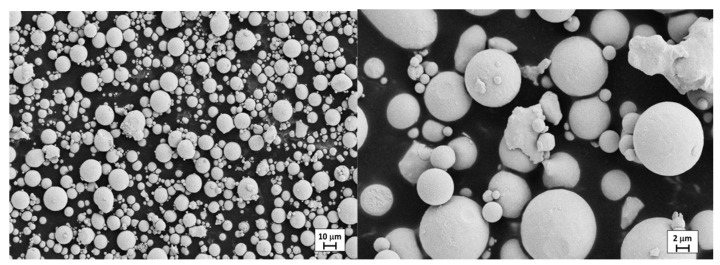
Powders morphology of CuSn12 alloy, SEM images.

Copper–Tin powder alloy produced by a gas-atomization process is considered in this work. The alloy’s nominal chemical composition (expressed in weight %) is 12% Sn and 88% Cu. The morphology characterization of the CuSn12 powder was carried out by means of high-resolution electronic scanning microscope (FE-SEM Zeiss LEO-1530). The shape of the powder appears generally spherical (Figure 3) with a size in the range between 5 and 20 µm (the average diameter is approximately equal to 11 µm). Moreover, ICP-MS analyses were carried out to detect the possible release of ions in water; results proved the absolute absence of any release of ions.

This powder was selected due to the complete lack of information in the literature about it and its relevant content of Cu. This latter material has been previously tested in the presence of methane, while results about its action in the presence of carbon dioxide are missing in the literature. For instance, Said et al. [31] observed the effect of Cu nanoparticles on a binary mixture made with methane (25 mol.%) and carbon dioxide (75 mol.%). They asserted that at low concentrations (0.1–0.2–0.3 wt.%), this additive acted as a kinetic promoter for the process. A similar trend was found in Pahlavanzadeh et al. (2019) [32] with the addition of low concentrations of CuO (0.1–0.3–0.5 wt.%).

Previous experiments led us to assert an inhibiting action of this compound on CO_2_ hydrates.

In the presence of CO_2_ as a guest compound, the results found in the literature were different from those observed during the experiments: Cu and CuO were defined as kinetic promoters. However, it might be due to the presence of CH_4_ in the initial mixture. In conclusion, Cu and CuO particles are known to act as kinetic promoters for methane hydrates, while the experimental evidence led us to affirm an opposite action on carbon dioxide hydrates.

The chemical composition, method of production, and current application of CuSn12 powder explained why this compound was selected for the present research [33].

### 2.3. Methods

Experiments were carried out in the presence of pure quartz porous sand. That porous medium was used to extend the gas hydrate formation to the whole volume and not only in that in correspondence with the gas–liquid interface. In addition, sand allowed us to distribute CuSn12 grains along the whole reactor. Conversely, the powder would settle on the bottom. Two tests were realized without the metallic powder previously described, while in the others, three different concentrations were used in order to investigate also the relation between hydrate formation and powder concentration. In particular, the following quantities were used: 50, 250, and 500 g. Throughout the manuscript, those quantities have been expressed as percentage of the whole amount of solid material present inside the reactor before hydrate formation or the sum of quartz sand and metallic powder. The three quantities indicated above correspond to 4.23 wt.%, 18.1 wt.%, and 30.66 wt.%. That choice was preferred to the others because the chemical additive was at the solid state and not at the liquid or gaseous, as additives are usually applied.

**Figure 4:**
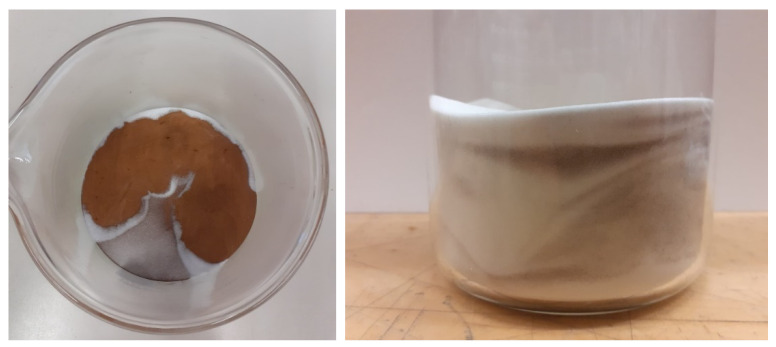
Quartz sand and metallic CuSn12 powder before and after their mixing.

Metallic powder was firstly mixed with silica sand until obtaining a satisfactory mixture; then, the reactor was filled with it and immediately after with water. In Figure 4, the mixture of sand and powder is shown.

The difference in density and granulometry made the achievement of a completely homogeneous phase difficult: metallic powder diffused elsewhere; however, its concentration was not completely uniform and, as it is possible to see in Figure 4 (at right), it assumed higher concentrations in some contained portions.

The chiller was regulated in order to generate an internal temperature about 1–3 °C inside the reactor. Gas injection occurred slowly in order to increase pressure by maintaining a constant gradient. In tests carried out with methane, the initial pressure was fixed at 54–56 bar, while in tests where carbon dioxide was involved, it ranged from 38 to 40 bar. The presence of feasible conditions immediately led to hydrate production, whose formation was proved by a constant pressure decrease. Initially, the temperature increased significantly due to heat production caused by water cages formation; then, it constantly decreased until reaching the thermal balance with the system. As soon as the pressure stabilized, the formation process was considered over. Following that, the chiller was switched off, and thermal energy was provided from the external to increase the temperature again and cause hydrate dissociation. The strategy adopted to increase the temperature during the dissociation phase was selected according to previous research, which proved that the experimental results produced in this way had high similarity with the equilibrium conditions widely described in the literature. In addition, further experiments were carried out where temperature increase was regulated from the external and a lower temperature gradient was established. The comparison between results reached in this way and those present in this article proved again the accuracy of the method here adopted. This latter phase was used to consider equilibrium values for both types of guest compounds in the presence of CuSn12 powder.

## 3. Results

As previously explained, methane and carbon dioxide hydrate formation were firstly tested in the presence of pure silica sand and then in the presence of three different concentration of CuSn12 powder: 4.23 wt.%, 18.1 wt.%, and 30.66 wt%. Hydrate compounds were formed and then left free to dissociate in order to produce equilibrium values. A comparison between the results here produced and equilibrium values present elsewhere in the literature was provided [34,35,36,37,38,39,40,41,42,43,44,45,46,47,48,49,50,51,52,53,54,55,56,57,58,59].

This section has been divided into three parts: firstly, methane hydrate formation tests are described and represented in pressure–temperature diagrams; then, a similar section is dedicated to tests involving carbon dioxide. Finally, the results produced with these two compounds were put together and compared in order to verify if the use of this CuSn12 powder produced similar effects in both cases or not.

### 3.1. Methane Hydrate Formation Tests

**Figure 5:**
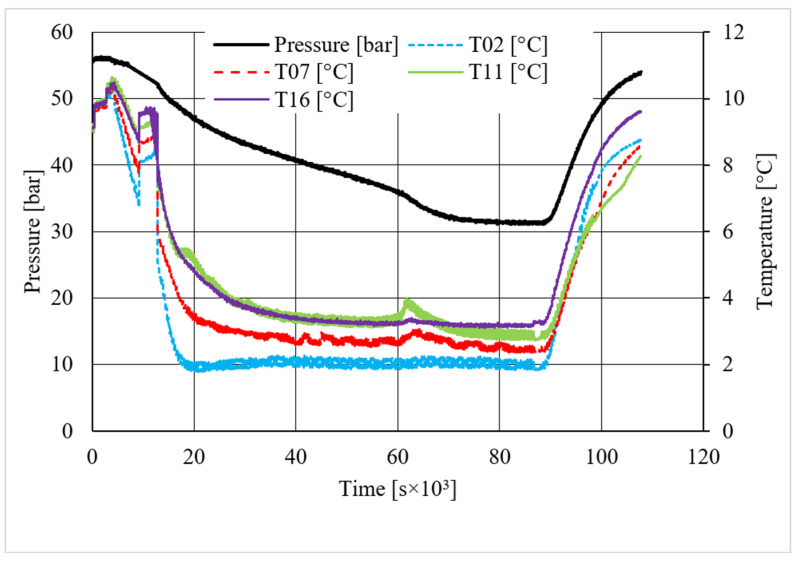
Pressure and temperature trends over time, measured for methane hydrate formation in the absence of CuSn12 powder.

**Figure 6:**
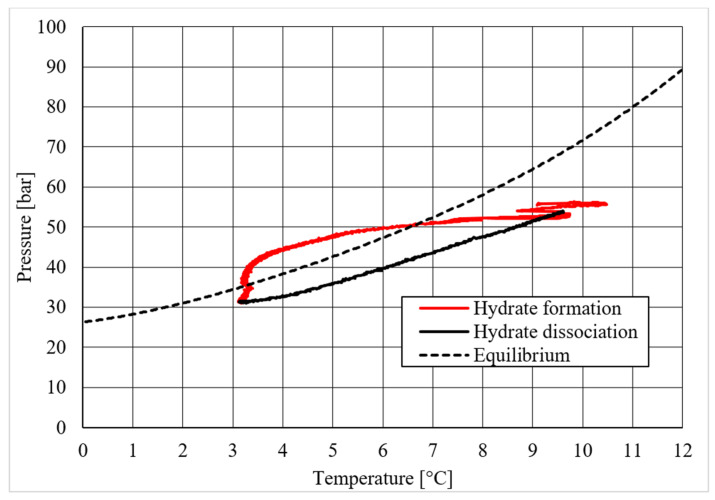
Pressure vs. temperature, describing methane hydrate formation in the absence of CuSn12 powder.

**Figure 7:**
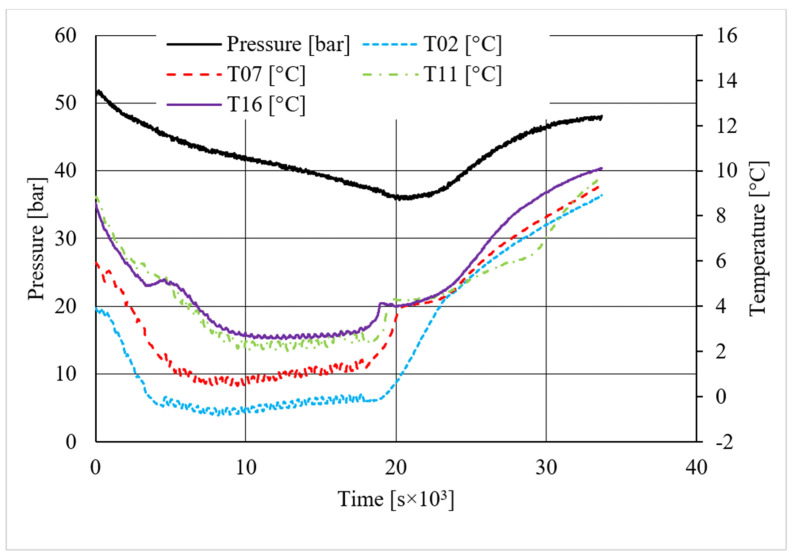
Pressure and temperature trends over time, measured for methane hydrate formation in the presence of 4.23 wt.% CuSn12 powder.

**Figure 8:**
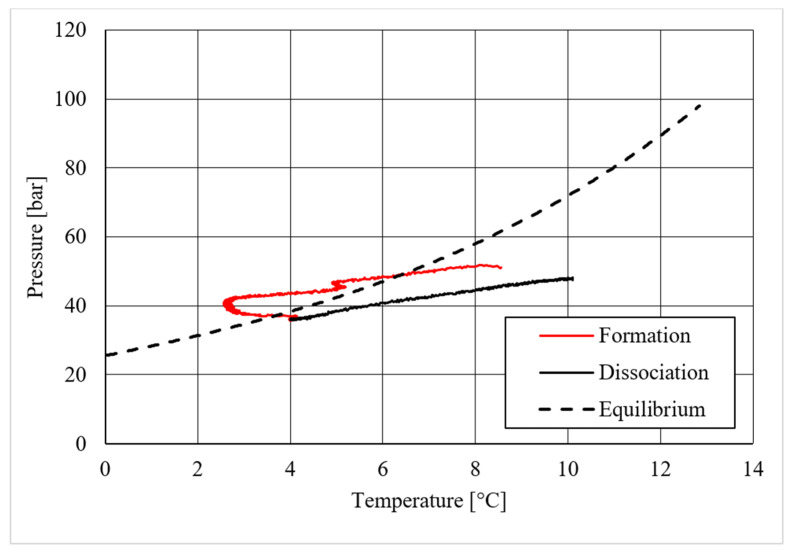
Pressure vs. temperature, describing methane hydrate formation in the presence of 4.23 wt.% CuSn12 powder.

**Figure 9:**
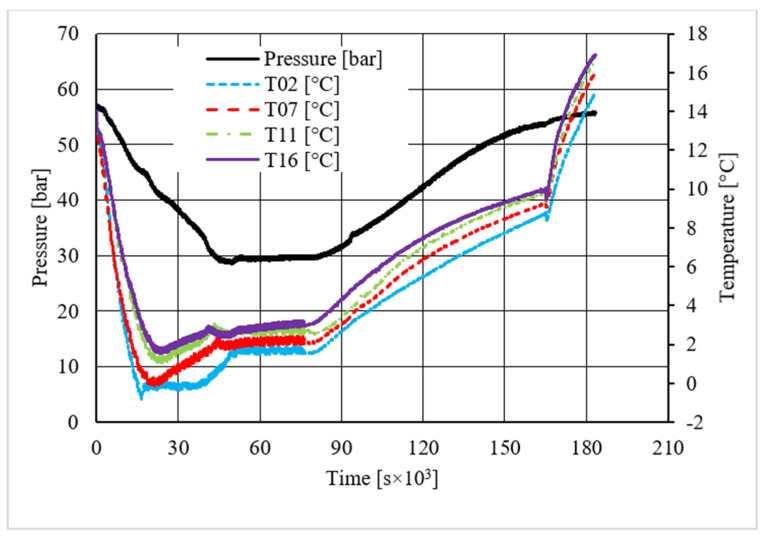
Pressure and temperature trends over time, measured for methane hydrate formation in presence of 18.01 wt% CuSn12 powder.

**Figure 10:**
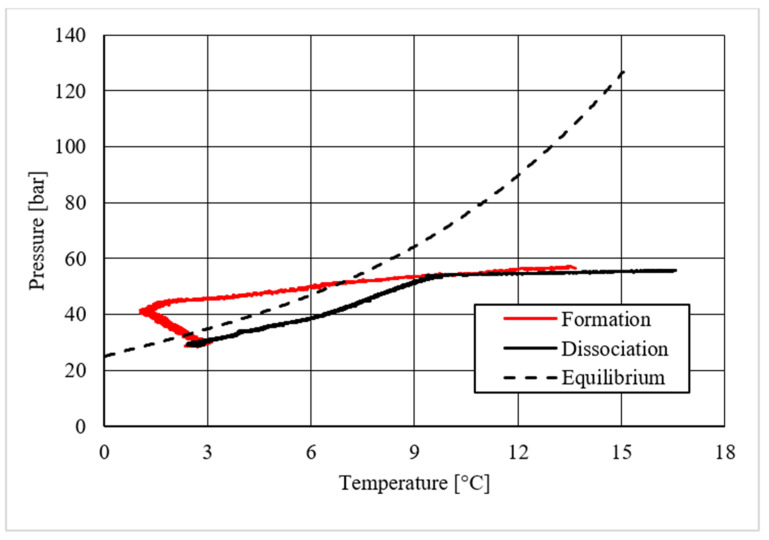
Pressure vs. temperature, describing methane hydrate formation in the presence of 18.01 wt.% CuSn12 powder.

**Figure 11:**
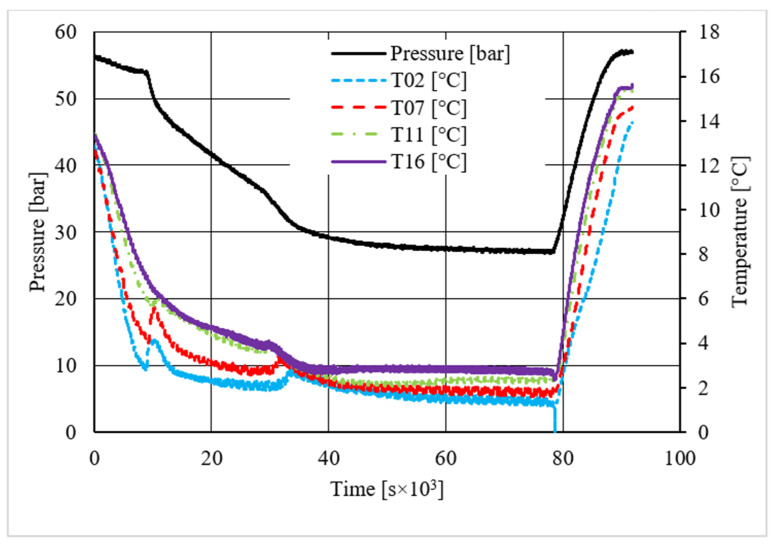
Pressure and temperature trends over time, measured for methane hydrate formation in the presence of 30.66 wt.% CuSn12 powder.

**Figure 12:**
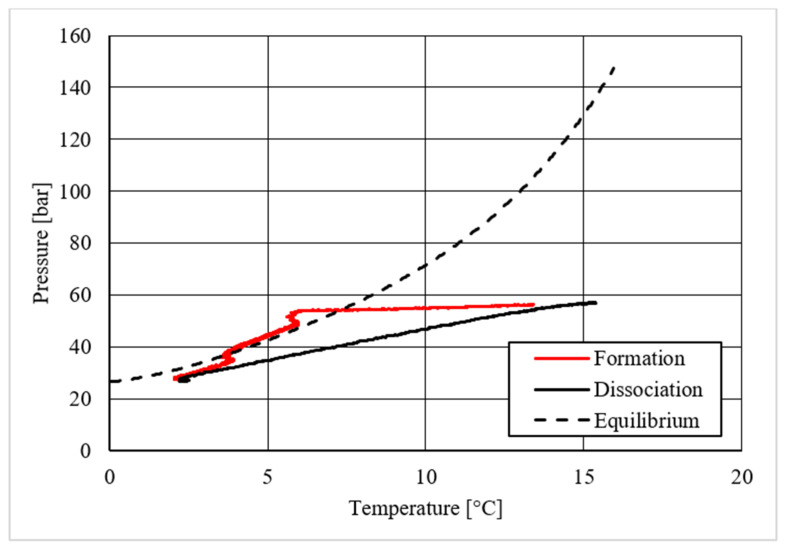
Pressure vs. temperature, describing methane hydrate formation in the presence of 30.66 wt.% CuSn12 powder.

Table 2 shows equilibrium values for methane hydrate both in the absence and in the presence of the CuSn12 powder previously described. According to the method present in the literature, those equilibrium values were taken during hydrate dissociation. In fact, the dissociation phase followed an almost linear trend and was less affected by external parameters, unlike hydrate formation, whose temperature trend suffered from the formation of new hydrate formation nuclei, which caused local and delayed temperature peaks as well as the tendency of the whole system to reach thermal balance with the surrounding experimental apparatus. Then, Figure 5, Figure 6, Figure 7, Figure 8, Figure 9, Figure 10, Figure 11 and Figure 12 describe the pressure and temperature trends over time and the pressure–temperature trend observed during tests involving methane. For each different concentration analyzed, one test is shown. In the first type of diagram, values measured by all thermocouples are shown in order to understand where hydrate formation mainly occurred and also to verify if secondary hydrate nuclei appeared during the hydrate massive growth phase. In diagrams where pressure was described as a function of temperature, this latter parameter was calculated as the average of values measured by each thermocouple.

The temperature trends over time revealed that hydrate formation mainly affected the lowest portion of the volume available for the process: the most significant variations in temperature, associated to the exothermic nature of such reactions, were observed with thermocouples T16 and T11. However, also, the remaining volume was involved, and several secondary peaks in temperature were observed with T07 and T02. In the first experiment shown, carried out without the CuSn12 powder, the first peak, or the main temperature variation immediately after hydrate formation occurs, was not verified as soon as the thermodynamic conditions made hydrate formation feasible; instead, it occurred with a certain delay in time. That aspect is commonly due to the stochastic behavior of the process. The corresponding diagram describing the pressure trends over time showed that any decrease occurred before such temperature variation. In the same experiment, a secondary peak in temperature was observed by all thermocouples, which made the pressure drop more intense. In the remaining part of the process, only thermocouple T11 measured further temperature peaks, which were less intense than the previous one. In every case, a variation in pressure reduction gradient was observed in the correspondence of all those peaks.

Differently from it, in the last test, where the CuSn12 powder was inserted inside the reactor, with a concentration equal to 30.66 wt.%, secondary and delayed peaks in temperature were observed in regions associated to T02 and T07, or near the top. As expected, those peaks were immediately followed by a more intense pressure drop than before.

The detection of nucleation sites with all thermocouples proved that methane well diffused in the whole reactor and, similarly, that hydrate formation mainly occurred in sand pores. The first information justified the choice of using a porous medium to produce an internal skeleton able to guarantee diffused hydrate formation. Conversely, only the gas–liquid interface would have been affected by the reaction. The sand pores created a lot of little gas–liquid interfaces inside them, in particular because methane was injected from the bottom; thus, it had pass through the entire medium before reaching the upper surface. Considering the time spent thoroughly mixing sand with CuSn12 powder, whose size is extremely lower than that of sand grains, and taking into account the results obtained in this experience (which will be described in the following lines), we believed that powder adhered to the sand grains, thus influencing hydrate formation both inside and outside sand pores.

Diagrams showing the relation existing between pressure and temperature provided useful information to understand whether that powder intervened or not in the formation process and, in positive cases, what effects it had. The first of those diagrams refers to hydrate formation carried out in the absence of CuSn12 powder. Here, the continuous black line, related to hydrate dissociation, remained completely below the equilibrium curve. It means that the particular silica-based porous medium used during experiments and previously described in Section 2 acted as a promoter for methane hydrate formation. More in-depth, such a promoting effect was observed to increase with temperature: in Figure 6, as in all other P-T diagrams, these two curves moved away from each other in the presence of higher temperature values. That deviation was also found in the presence of powder; however, in this latter case, further considerations were required.

Diagrams describing tests made with concentrations of CuSn12 powder of 4.23 and 18.01 wt.% led to results very similar to those reached in its absence: values of hydrate dissociation were slightly shifted to lower pressures and higher temperature regions, proving again that milder conditions were needed to form hydrate. However, no meaningful variation in the process condition was found. The addition of powder, with the concentrations reported above, did not affect hydrate formation, neither inhibiting nor promoting it. The horizontal trend present in Figure 10 is not particularly meaningful: once the maximum pressure value was reached, the dissociation diagram could only assume that horizontal trend. The values reported in Table 2 provided additional confirmation, as all the values were similar between each other, and in all cases, the observed promoting effect can be completely associated with sand properties.

A completely different behavior was observed in the last experiment, where the CuSn12 powder was used with a concentration equal to 30.66 wt.%. For temperature values higher than 3.8–4.0 °C, the pressure and temperature conditions required to form hydrate become significantly milder than in previous experiments. Thus, with this last concentration, the powder acted as a promoter for methane hydrate formation. As in previous tests, the distance between the ideal equilibrium curve and experimental values increased with temperature. Table 2 confirmed the tendency as soon described.

**Figure 13:**
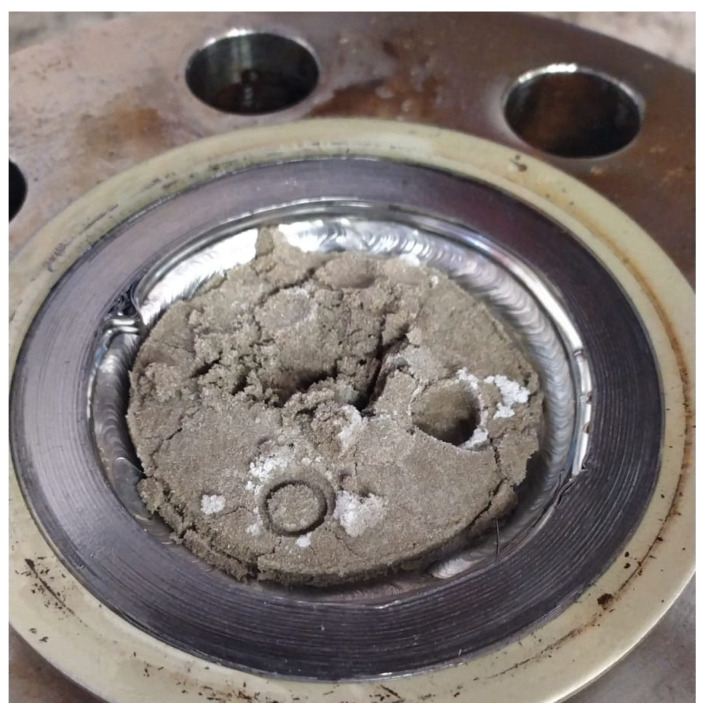
As soon as the test with 30.66 wt.% CuSn12 powder finished, the reactor was opened, and the medium was found to be much more compact than in the absence of powder.

Considering that the promoting effect was observed only in the presence of a certain concentration, it cannot be motivated with the chemical composition of that powder or geometrical characteristics of its grains. As it is possible to see in Figure 13, such powder acted as a cementing element for sand grains, thus hindering methane escape.

In addition, that powder surely increased the heat transfer rate inside the porous medium, thus giving a further contribution to hydrate formation.

### 3.2. Carbon Dioxide Hydrate Formation Tests

**Figure 14:**
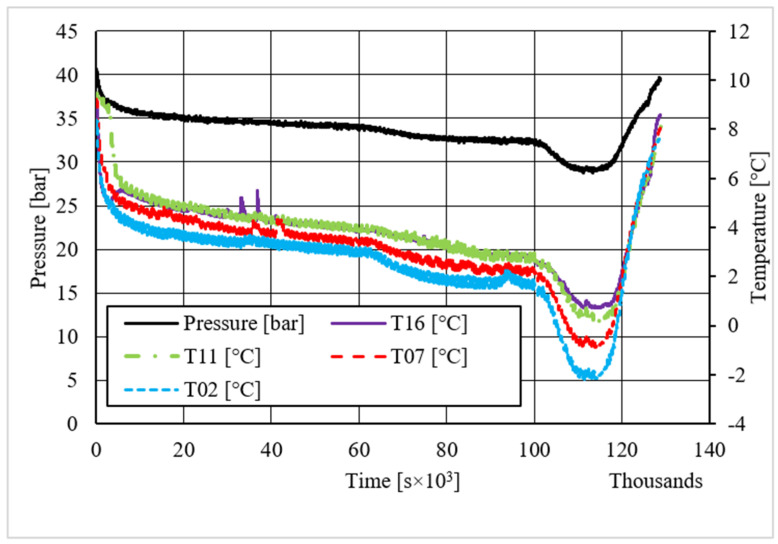
Pressure and temperature trends over time, measured for carbon dioxide hydrate formation in the absence of CuSn12 powder.

**Figure 15:**
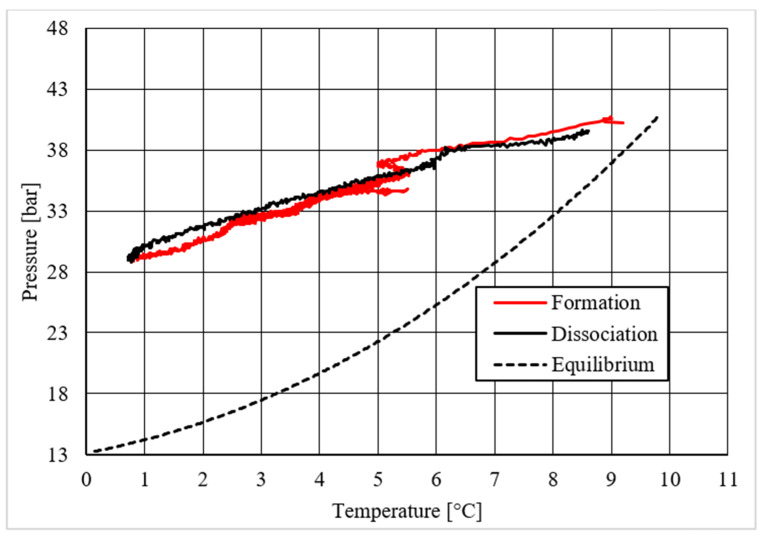
Pressure vs. temperature, describing carbon dioxide hydrate formation in the absence of CuSn12 powder.

**Figure 16:**
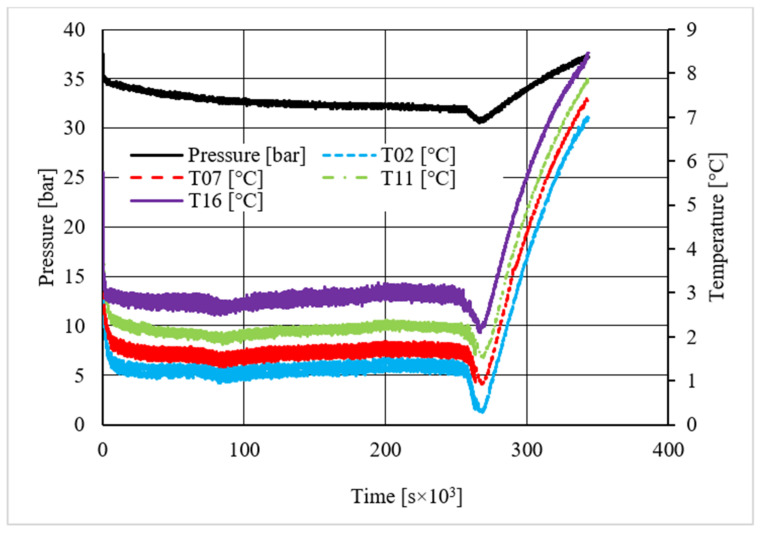
Pressure and temperature trend over time, measured for carbon dioxide hydrate formation in the presence of 4.23 wt.% CuSn12 powder.

**Figure 17:**
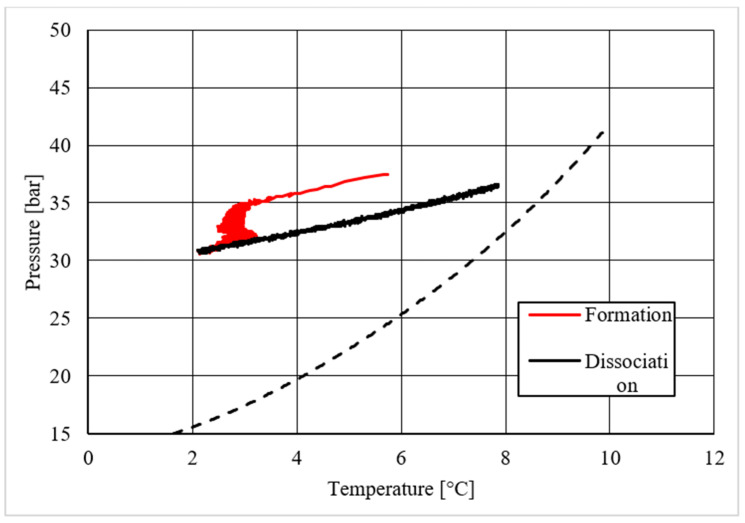
Pressure vs. temperature, describing carbon dioxide hydrate formation in the presence of 4.23 wt.% CuSn12 powder.

**Figure 18:**
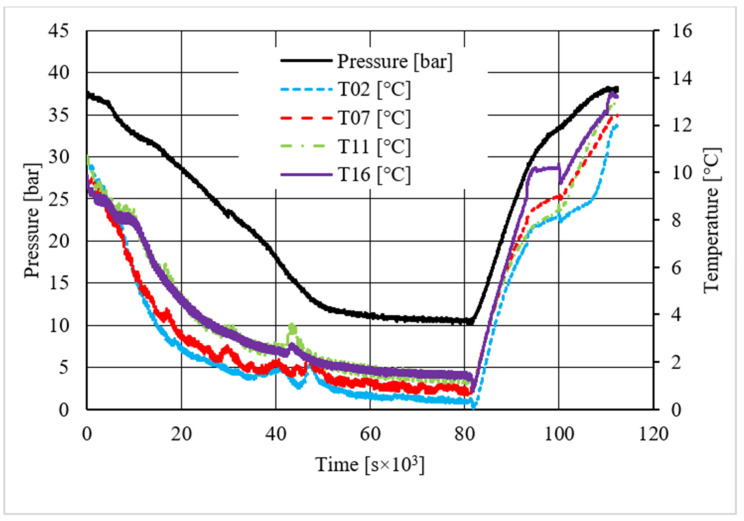
Pressure and temperature trend over time, measured for carbon dioxide hydrate formation in the presence of 18.01 wt.% CuSn12 powder.

**Figure 19:**
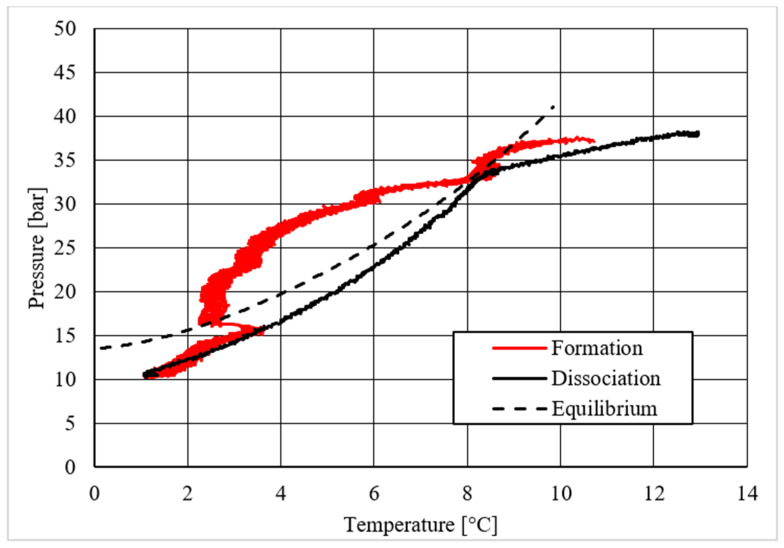
Pressure vs. temperature, describing carbon dioxide hydrate formation in the presence of 18.01 wt.% CuSn12 powder.

**Figure 20:**
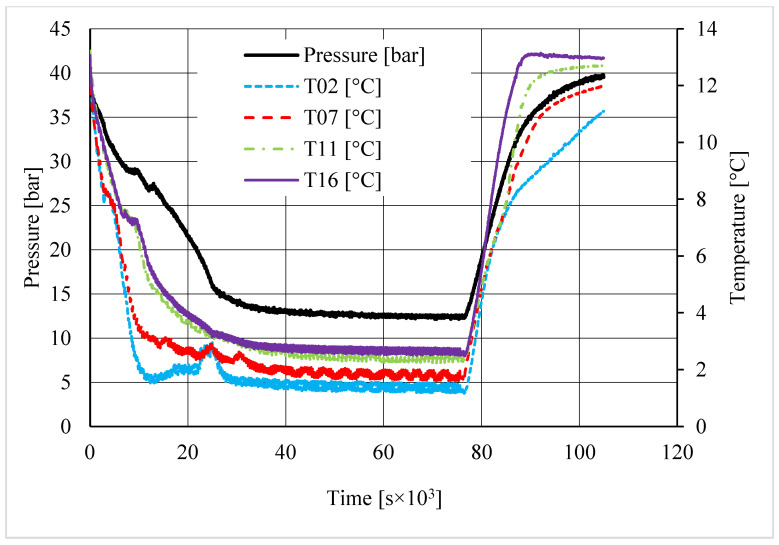
Pressure and temperature trend over time, measured for carbon dioxide hydrate formation in the presence of 30.66 wt.% CuSn12 powder.

**Figure 21:**
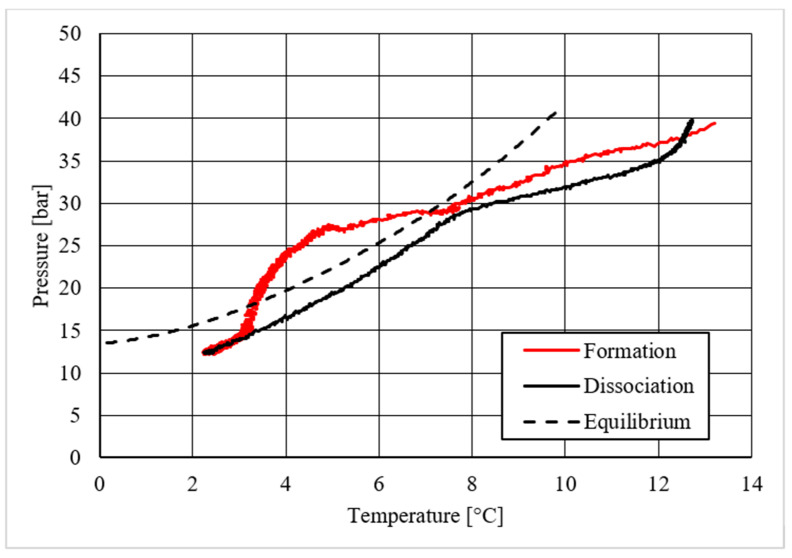
Pressure vs. temperature, describing carbon dioxide hydrate formation in the presence of 30.66 wt.% CuSn12 powder.

As in the previous section, a table was inserted to describe the hydrate equilibrium values measured in all tests (see Table 3); then, one experiment for each different concentration of CuSn12 (or 0, 4.23, 18.1, and 30.66 wt.%) powder was described by showing the trend over time of its thermodynamic parameters and with a corresponding pressure–temperature diagram. Those diagrams are visible in Figure 14, Figure 15, Figure 16, Figure 17, Figure 18, Figure 19, Figure 20 and Figure 21. All experiments were carried out by fixing an initial internal pressure in the range of 37–40 bar, thus lower than the corresponding range established for methane hydrate formation tests, due to the milder conditions required by systems involving carbon dioxide to form hydrate.

As for methane, also, carbon dioxide hydrate formation mainly affected the portion of internal volume corresponding to thermocouples T11 and T16, whose trend remained higher than other devices in all tests.

Secondary temperature peaks were observed in almost all experiments. Figure 14 shows the temperature and pressure trend over time measured in the absence of CuSn12 powder: thermocouple T11 described an initial peak more extended than the other devices. Then, T16 and T07 registered two secondary peaks, which occurred contemporarily. However, those events are independent from each other: thermocouple T11, which is inserted between T16 and T07, did not register any variation, proving that thermocouples revealed two different nucleation sites, which occurred at the same time.

Conversely to other tests, in the presence of 18.01 wt.% CuSn12 powder, the reaction started near the top of the reactor or in the correspondence of T02 and T07; then, the process involved the whole reactor, and the other two thermocouples started measuring temperature values higher than the others. In the same test, T11 also showed a secondary peak, which was associated to the formation of a new hydrate nucleation site. A similar trend was finally observed in the last tests, where the concentration powder was about 30.66 wt.%. In addition, here, secondary peaks were verified; however, they belonged to thermocouples T02 and T07. It means that hydrate formation firstly interested the lowest portion of the reactor and, in the following period, it involved the whole volume.

A first comparison between methane and carbon dioxide hydrate formation, based on pressure and temperature trend over time, did not reveal any substantial difference: hydrate formation was found to occur elsewhere, even if it mainly affected the regions corresponding to T11 and T16, and the formation of secondary nucleation sites occurred in almost all experiments, despite the quantity of CuSn12 powder used.

On the contrary, important differences were noticed in the diagrams describing the pressure trend as a function of temperature.

Despite what was observed for methane, the porous medium acted as an inhibitor for carbon dioxide hydrate formation. Figure 15 clearly shows that values describing hydrate dissociation remained above the ideal equilibrium curve during the whole test. Additionally for this guest compound, the formation conditions became milder with increasing temperature values.

The same porous medium acted as a promoter for methane hydrate, while it inhibited carbon dioxide hydrate formation. The main reason of such a difference can be found in sand grains and, in particular, in their size.

Borchardt et al. [60] explained how hydrate growth in inner pores is completely different from that in bulk water: the lattice formed in micro and meso pores is generally smaller, due to confinement effects. For methane, nano hydrate structures were proved to grow in micro pores, with a stoichiometry about one molecule of methane trapped every two molecules of water involved [61]. Both guest compounds studied in this work usually form sI hydrate; however, the CO_2_ molecule is larger than that of CH_4_, thus, it may encounter more difficulties in forming hydrate inside inner pores.

The addition of a small amount of CuSn12 powder to the porous medium did not cause any significant change: the hydrate dissociation values remained above the ideal equilibrium curve. However, in this latter case, the dissociation values were slightly shifted to lower pressures than in the absence of powder, thus proving a little promoting effect. This can be easily seen in Table 3, where the results measured during each test are shown and compared.

The introduction of higher quantities of powder inside the reactor led to completely different results. Hydrate formation and dissociation values became significantly milder, until moving below the ideal equilibrium curve. In particular, the dissociation values remained completely below it. The relation with temperature verified in previous experiments was observed also in those cases, where it become even more pronounced. Few differences were found between hydrate formation in the presence of 18.01 wt.% and 30.66 wt.% CuSn12 powder: Table 3 proved again that the greater usage of such powder, or 30.66 wt.%, led to milder dissociation values.

These two concentrations corresponded to the introduction of respectively 250 g and 500 g of powder inside the reactor. In the latter case, the usage of a double quantity of powder only produced a weak effect rather than further promoting action. Consequently, further additions were not considered useful to improve that promoting effect and were not taken into account.

As for methane hydrate, the results reached in this experience were not expected. Very few data about hydrate formation in the presence of metal alloys are currently present in the literature and, in all cases, copper is considered an inhibitor for the process [62,63].

Finally, based on previous research studies [64], a brief description of the formation process is here proposed. This process can be mainly distinguished in two phases: hydrate nucleation and massive growth phases. These two processes can be easily observed in the pressure–temperature diagrams, because they usually assume different trends. For instance, Figure 12, Figure 15 and Figure 19 well show it. During nucleation, pressure decreased slowly due to the contemporary formation and dissociation of hydrates. The main pressure drop occurred during the growth phase, during which the pressure decreased drastically even if the temperature did not show equally relevant variations. This phenomenon happened because the hydrate crystals inside the reactor reached the so-called “critical size” and the spontaneous dissociation of them finished, while they continued their growth. This latter aspect can be explained with the Labile Cluster Theory [1,64], which describes the formation of hydrate nuclei during the first phase, or hydrate nucleation.

According to hydrate nucleation, liquid water molecules initially absorb the gaseous molecules, form primordial clusters, which are composed by a guest molecule and 20–24 water molecules, and finally generate the first unstable 5^12^ cages. These structures may continue their growth, via collision with other structures, or dissociate. When vertices are shared during collision, small cubic sI units are formed. Conversely, when faces are shared, small cubic sII cages are produced. In addition, these structures may dissociate or continue their growth. This latter phase continues until the hydrate nuclei reach the critical size; then, massive growth occurs. In the figures previously mentioned, the difference between nucleation and massive growth is well visible: nucleation is less expectable, because it depends on numerous variables and consequently consists of a stochastic process. Conversely, the massive growth appears with a drastic decrease in pressure, which may occur as soon as the equilibrium conditions are reached (obviously, the induction time must be considered, because the transition phase, during which the system goes through the so-called metastable region, cannot be neglected). Such characteristics are particularly helpful in defining the entity of the inhibiting/promoting effect associated to the presence of CuSn12 particles.

### 3.3. Direct Comparison between Results Reached with Methane and Carbon Dioxide

In this concluding section, the dissociation values measured during all the experiments, both in the presence of methane or carbon dioxide as a guest compound, were shown in a single diagram in order to provide a direct comparison among those values and, if possible, suggest potential applications.

**Figure 22:**
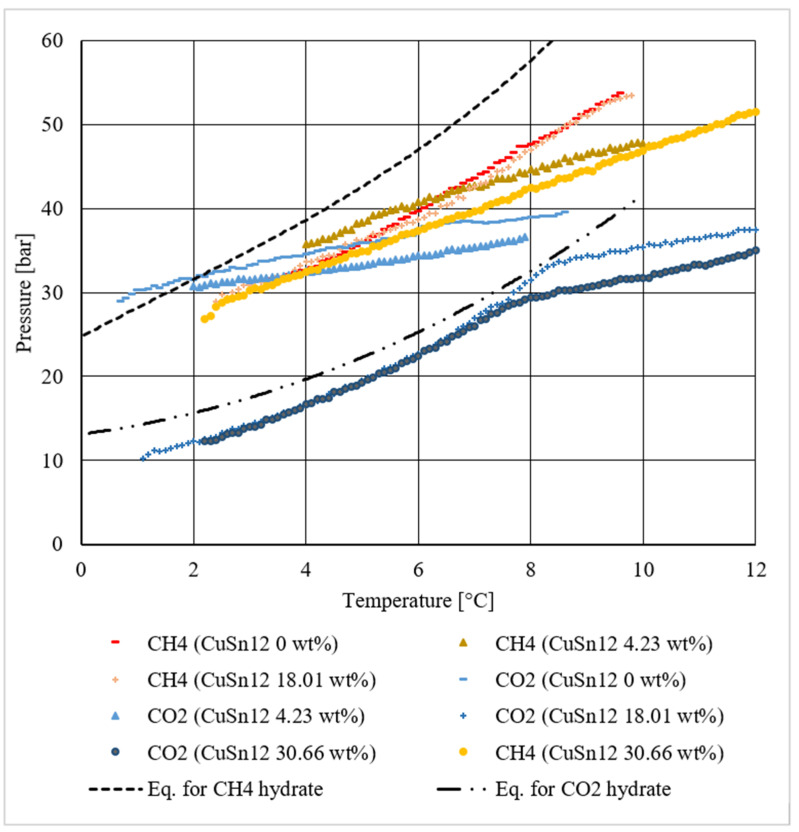
Hydrate dissociation conditions measured during all tests and compared among each other.

Figure 22 shows all the results produced in this work, together with ideal equilibrium curves related to both compounds and those already shown in previous diagrams.

In that figure, the two dotted lines have been used to indicate the ideal methane (the higher) and carbon dioxide hydrate equilibrium (the lower). Warm colors were used to describe experiments involving methane, while cold colors described tests made with carbon dioxide.

As previously explained, in all the tests, methane hydrate formation occurred at milder conditions than equilibrium, while a high dosage of CuSn12 powder was necessary to reach a similar result in the presence of carbon dioxide. In Figure 22, different indicators were used to represent tests made with a certain powder concentration. The quartz-based porous medium used drastically reduced the distance existing between the formation conditions of hydrate containing these two gaseous compounds. In the presence of temperatures lower than 4.5–4.0 °C, CO_2_ hydrate required more severe conditions than CH_4_ hydrate. The introduction of 4.23 wt.% CuSn12 powder did not cause significant variations, while for higher concentrations, values associated to CO_2_ hydrate formation drastically dropped below the ideal equilibrium curve. Similar variations in tests involving methane were found only with 30.66 wt.% powder, and according to CO_2_-based tests, they become more and more evident with temperature increase.

Based on the results discussed in this work, the present metallic powder might be used for gas storage. The maximum concentration tested was about 30.66 wt.%, the remaining solid medium consisted of pure quartz sand and was used to guarantee a homogeneous diffusion CuSn12 particles along the whole reactor. With appropriate treatments, a similar powder might be used to directly build a metallic high-porous lattice, thus allowing the use of higher concentrations and optimization of the promoting effect. In that sense, the powder accurately described in those pages may contribute to improving the methane storage capacity of tanks and cylinders, thus increasing the energy density of their content.

## 4. Conclusions

The present work allowed us to investigate methane and carbon dioxide hydrate formation in the presence of a porous pure quartz medium and a powder consisting of a CuSn12 metallic alloy. Experiments were carried out with the aim of establishing if this latter material was able to intervene in hydrate formation and dissociation or not and, in positive cases, defining the entity and modalities of its action. A small-scale reactor was used to produce hydrate, firstly in the absence of powder and then by adding it in different concentrations of 4.23, 18.01, and 30.66 wt.%.

The first experimental evidence was provided by the porous medium, which acted as a promoter for methane hydrate, while it inhibited the process in the presence of carbon dioxide. That behavior was related to the geometrical properties of sand grains and their pores.

In both cases, the CuSn12 powder was found to promote hydrate formation. When methane was used as a guest compound, that promoting effect was observed only with a concentration about 30.66 wt.%; however, it was significant and moved hydrate dissociation values to milder thermodynamic conditions. The same occurred in the presence of carbon dioxide with the only difference that also a lower concentration, or 18.01 wt.%, promoted hydrate formation. In this latter case, while sand properties initially fixed conditions of hydrate dissociation above the ideal equilibrium, the involvement of powder brought those values below it.

Based on results reached in this work, it is expected that higher concentrations may further lead to milder dissociation conditions. From here, there is the possibility, which clearly needs to be further researched, to use such powder to build, with the auxilium of 3D print technologies, high-porous lattice in apposite tanks, to improve the gas storage efficiency and the density of energy stored per unit of volume. Further research will be focused on investigating why such metallic powder acted as a promoter for hydrate formation. In particular, analyses on water composition after experiments will be done to detect potential ions release in water. Alongside water, the solid medium will be further investigated as well to determine its variation in thermal conductivity due to the addition of such metallic powder.

## Figures and Tables

**Table 1:** Geometric and volume specification about the lab-scale reactor.

Board Height Internal Height	21 22.1	cm cm	Internal Pipe Volume Volume of Intake Pipes	1 18	cm^3^ cm^3^
Depth from the edge	18.5	cm	Gas volume from the high edge	90	cm^3^
Depth edge to the network	18.3	cm	Total volume of free gas	109	cm^3^
Weld thickness	1.1	cm	Internal reactor volume	949	cm^3^
Thickness from the edge	1	cm	Total reactor volume	950	cm^3^
Internal diameter	7.4	cm	–	–	–

**Table 2:** Equilibrium values for methane hydrate in the presence of CuSn12 powder, analyzed with four different concentrations.

Temperature [°C]	0 [wt.%]	4.23 [wt.%]	18.1 [wt.%]	30.66 [wt.%]
2.2				26.86
2.3				27.19
2.4			28.93	28.32
2.5			29.72	28.84
2.6			29.46	29.1
2.7			29.99	29.3
2.8			30.34	29.57
2.9			30.86	29.68
3			30.89	30.25
3.1	31.25		31.34	30.47
3.2	31.28		31.29	30.35
3.3	31.34		31.14	30.8
3.4	31.58		31.57	30.91
3.5	31.56		31.91	31.25
3.6	31.9		31.91	31.56
3.7	32.12		32.13	31.67
3.8	32.32		32.66	31.91
3.9	32.52		33.15	32.13
4	32.64	35.78	33.74	32.48
4.1	32.98	35.91	33.68	32.7
4.2	33.11	36.01	33.87	32.76
4.3	33.67	36.36	34.32	33.31
4.4	33.87	36.36	34.4	33.47
4.5	34.31	36.7	34.41	33.63
4.6	34.45	37.04	35.2	33.88
4.7	34.76	37.43	35.72	34.19
4.8	35.25	37.46	35.65	34.68
4.9	35.51	38.23	36.26	34.63
5	35.87	38.32	36.12	34.85
5.1	36.12	38.58	36.44	34.89
5.2	36.65	39.14	36.94	35.53
5.3	36.89	39.34	36.8	35.56
5.4	37.41	39.54	37.23	35.88
5.5	37.87	39.77	37.42	36.07
5.6	38.08	39.9	37.71	36.36
5.7	38.73	40.24	38	36.8
5.8	38.91	40.09	38.31	36.97
5.9	39.37	40.26	38.31	37.11
6	39.68	40.82	38.68	37.34
6.1	40.04	40.88	38.94	37.56
6.2	40.35	41.41	39.37	37.95
6.3	41.02	41.1	39.43	38.09
6.4	41.33	41.38	40.33	38.34
6.5	41.9	41.7	40.34	38.63
6.6	42.39	42.13	40.62	38.86
6.7	42.48	42.21	41.37	39.12
6.8	43.02	42.46	41.29	39.17
6.9	43.27	42.57	42.34	39.48
7	43.54	42.73	42.57	39.69
7.1	44.08	42.61	42.92	39.79
7.2	44.26	42.82	43.14	40.27
7.3	44.84	43.21	43.81	40.56
7.4	45.35	43.56	44.38	40.79
7.5	45.62	43.52	44.53	40.95
7.6	46	43.61	44.89	41.03
7.7	46.61	43.71	45.81	41.46
7.8	47.39	44.26	46.16	41.76
7.9	47.35	44.23	46.74	42.19
8	47.58	44.7	47.05	42.48
8.1	47.84	44.41	47.54	42.4
8.2	48.29	45.01	47.85	42.71
8.3	48.64	44.74	48.3	42.8
8.4	48.9	45.31	48.6	43.05
8.5	49.18	45.45	49.26	43.52
8.6	49.55	46.07	49.8	43.62
8.7	50.06	45.6	50.2	43.65
8.8	50.63	46.29	50.33	44.19
8.9	51.21	46.16	51.08	44.46
9	51.53	46.51	51.08	44.53
9.1	51.81	46.74	51.57	44.44
9.2	52.34	46.65	51.89	44.98
9.3	52.65	46.81	52.47	45.37
9.4	52.87	47.19	52.77	45.59
9.5	53.36	46.96	53	45.86
9.6	53.69	47.25	53.14	46.1
9.7		47.38	53.32	46.09
9.8		47.77	53.45	46.42
9.9		47.83		46.63
10		47.77		46.89
10.1		47.54		47.37
10.2				47.51
10.3				47.64
10.4				47.96
10.5				48.26
10.6				48.31
10.7				48.44
10.8				48.83
10.9				48.97
11				49.3
11.1				49.46
11.2				49.73
11.3				49.99
11.4				50.08
11.5				50.44
11.6				50.73
11.7				51.09
11.8				51.12
11.9				51.36
12				51.57
12.1				51.68
12.2				51.95
12.3				52.26
12.4				52.22
12.5				52.45
12.6				52.88
12.7				53.14
12.8				53.37
12.9				53.16
13				53.35
13.1				53.91
13.2				53.94
13.3				54.08
13.4				54.39
13.5				54.56
13.6				54.78
13.7				54.76
13.8				55.03
13.9				55.34
14				55.44
14.1				55.58
14.2				55.59
14.3				55.81
14.4				55.88
14.5				56.18
14.6				56.45
14.7				56.41
14.8				56.57
14.9				56.67
15				56.57
15.1				56.54
15.2				56.91
15.3				56.65
15.4				56.87

**Table 3:** Equilibrium values for carbon dioxide hydrate in the presence of CuSn12 powder, analyzed with four different concentrations.

Temperature [°C]	0 [wt.%]	4.23 [wt.%]	18.1 [wt.%]	30.66 [wt.%]
0.7	28.97			
0.8	29.24			
0.9	29.73			
1	30.25			
1.1	30.22		10.24	
1.2	30.34		10.73	
1.3	30.78		11.14	
1.4	30.56		11.04	
1.5	30.91		11.24	
1.6	31.09		11.47	
1.7	31.36		11.75	
1.8	31.55		11.78	
1.9	31.55		12.08	
2	31.42	30.78	12.36	
2.1	31.98	30.68	12.16	
2.2	31.89	30.88	12.58	12.34
2.3	32.23	31.07	12.62	12.31
2.4	32.4	30.99	12.85	12.43
2.5	32.43	31.25	13.22	12.85
2.6	32.52	31.28	13.39	13.16
2.7	32.92	31.25	13.74	13.3
2.8	32.84	31.56	13.77	13.29
2.9	32.79	31.61	14.08	13.75
3	33.22	31.49	14.06	14.01
3.1	33.31	31.56	14.53	14.04
3.2	33.68	31.69	14.64	14.26
3.3	33.66	31.78	14.7	14.83
3.4	33.86	31.87	15.1	14.91
3.5	34	31.8	15.4	15.11
3.6	34.15	31.99	15.67	15.45
3.7	34.22	32.13	15.82	15.75
3.8	34.32	32.21	15.94	15.97
3.9	34.32	32.21	16.42	16.22
4	34.52	32.52	16.6	16.73
4.1	34.65	32.43	16.91	16.76
4.2	34.88	32.59	17.03	17.3
4.3	34.98	32.74	17.3	17.3
4.4	35.28	32.76	17.88	17.41
4.5	35.26	32.79	17.97	18.19
4.6	35.4	32.92	18.19	18.16
4.7	35.26	32.96	18.76	18.49
4.8	35.56	33.1	18.81	18.76
4.9	35.72	33.09	19.07	18.94
5	35.82	33.19	19.51	19.28
5.1	35.87	33.31	19.86	19.57
5.2	35.87	33.49	19.86	19.91
5.3	36.13	33.62	20.2	20.31
5.4	36.32	33.63	20.94	20.49
5.5	36.35	33.66	21.12	20.76
5.6	36.31	33.87	21.31	21
5.7	36.54	33.98	21.57	21.53
5.8	36.72	34	22.15	21.8
5.9	36.79	34.26	22.43	22.07
6	37.42	34.37	22.65	22.46
6.1	37.8	34.45	23.03	23.11
6.2	37.91	34.45	23.46	23.31
6.3	37.85	34.59	23.8	23.45
6.4	38.32	34.67	24.33	24.01
6.5	38.19	35.07	24.65	24.12
6.6	38.28	35.18	24.91	24.72
6.7	38.32	35.07	25.58	25
6.8	38.39	35.29	25.96	25.37
6.9	38.39	35.32	26.4	25.81
7	38.35	35.42	26.97	25.98
7.1	38.35	35.55	27.46	26.72
7.2	38.23	35.56	27.76	26.87
7.3	38.36	35.76	28.36	27.48
7.4	38.38	35.88	28.54	27.58
7.5	38.44	36	28.71	28.1
7.6	38.61	36.17	29.18	28.47
7.7	38.59	36.13	29.99	28.67
7.8	38.67	36.38	30.51	28.85
7.9	38.78	36.57	31.07	29.12
8	38.93		31.42	29.37
8.1	38.92		32.06	29.37
8.2	39.07		32.53	29.55
8.3	39.03		33.02	29.59
8.4	39.04		33.31	29.9
8.5	39.34		33.64	30.21
8.6	39.51		33.53	30.25
8.7			33.87	30.23
8.8			34.17	30.33
8.9			34.11	30.56
9			34.41	30.68
9.1			34.25	30.79
9.2			34.35	30.91
9.3			34.53	31.12
9.4			34.87	31.08
9.5			34.9	31.32
9.6			34.96	31.56
9.7			34.92	31.61
9.8			35.21	31.65
9.9			35.41	31.69
10			35.35	31.75
10.1			35.74	31.69
10.2			35.65	32.17
10.3			35.63	32.22
10.4			35.94	32.4
10.5			35.8	32.55
10.6			36.14	32.76
10.7			36.14	33
10.8			36.32	32.9
10.9			36.36	33.32
11			36.36	33.29
11.1			36.62	33.18
11.2			36.73	33.4
11.3			36.88	33.63
11.4			36.79	33.86
11.5			36.88	34.09
11.6			37.11	34.19
11.7			37.41	34.41
11.8			37.41	34.41
11.9			37.43	34.77
12			37.47	35.08
12.1			37.6	35.34
12.2			37.61	35.57
12.3			37.94	36.01
12.4			37.99	36.66
12.5			37.91	37.19
12.6			37.95	38.89
12.7			37.99	39.67
12.8			37.93	
12.9			38.07	
13			38.2	

## Data Availability

The data presented in this study are available on request from the corresponding author.
